# STC-UNet: renal tumor segmentation based on enhanced feature extraction at different network levels

**DOI:** 10.1186/s12880-024-01359-5

**Published:** 2024-07-19

**Authors:** Wei Hu, Shouyi Yang, Weifeng Guo, Na Xiao, Xiaopeng Yang, Xiangyang Ren

**Affiliations:** 1 School of Electrical and Information Engineering of Zhengzhou University, Zhengzhou, China; 2 Faculty of Engineering, Huanghe Science and Technology University, Zhengzhou, China; 3 Medical 3D Printing Center of the First Affiliated Hospital of Zhengzhou University, Zhengzhou, China

**Keywords:** Renal tumor segmentation, U-Net, Selective kernel, Vision transformer, Coordinate attention

## Abstract

Renal tumors are one of the common diseases of urology, and precise segmentation of these tumors plays a crucial role in aiding physicians to improve diagnostic accuracy and treatment effectiveness. Nevertheless, inherent challenges associated with renal tumors, such as indistinct boundaries, morphological variations, and uncertainties in size and location, segmenting renal tumors accurately remains a significant challenge in the field of medical image segmentation. With the development of deep learning, substantial achievements have been made in the domain of medical image segmentation. However, existing models lack specificity in extracting features of renal tumors across different network hierarchies, which results in insufficient extraction of renal tumor features and subsequently affects the accuracy of renal tumor segmentation. To address this issue, we propose the Selective Kernel, Vision Transformer, and Coordinate Attention Enhanced U-Net (STC-UNet). This model aims to enhance feature extraction, adapting to the distinctive characteristics of renal tumors across various network levels. Specifically, the Selective Kernel modules are introduced in the shallow layers of the U-Net, where detailed features are more abundant. By selectively employing convolutional kernels of different scales, the model enhances its capability to extract detailed features of renal tumors across multiple scales. Subsequently, in the deeper layers of the network, where feature maps are smaller yet contain rich semantic information, the Vision Transformer modules are integrated in a non-patch manner. These assist the model in capturing long-range contextual information globally. Their non-patch implementation facilitates the capture of fine-grained features, thereby achieving collaborative enhancement of global–local information and ultimately strengthening the model’s extraction of semantic features of renal tumors. Finally, in the decoder segment, the Coordinate Attention modules embedding positional information are proposed aiming to enhance the model’s feature recovery and tumor region localization capabilities. Our model is validated on the KiTS19 dataset, and experimental results indicate that compared to the baseline model, STC-UNet shows improvements of 1.60%, 2.02%, 2.27%, 1.18%, 1.52%, and 1.35% in IoU, Dice, Accuracy, Precision, Recall, and F1-score, respectively. Furthermore, the experimental results demonstrate that the proposed STC-UNet method surpasses other advanced algorithms in both visual effectiveness and objective evaluation metrics.

## Introduction

Renal tumors, as a prevalent affliction in urology, exhibit an escalating incidence trend year by year. Compared to other tumors, early diagnosis of renal tumors is challenging, often resulting in patients reaching advanced stages by the time symptoms are identified, leading to a higher mortality rate. Additionally, the diverse array of renal tumor types presents significant differences in treatment response and prognosis. Hence, precise segmentation of renal tumors in medical image analysis holds paramount importance, providing crucial information for tumor assessment, treatment planning, and prognosis determination.

With the development of artificial intelligence technology, more and more machine learning methods, including deep learning methods, are being applied to the biomedical field [1]. Chandrasekar et al. [2] consider the limited possibility of drug testing in the pregnant population and use various algorithms such as K-Nearest Neighbors (KNN), Support Vector Classifier (SVC), and Multi-Layer Perceptron (MLP) to predict the fate of drugs crossing the placental barrier, achieving good predictive results. Ansari et al. [3] discuss the effective method of using existing knowledge to understand and predict the effects of drugs on neurological diseases. By accurately predicting using machine learning models, drug candidates that can be repurposed for neurological diseases are effectively identified. After that, machine learning and deep learning models are used by Ansari et al. [4] to analyze twelve-lead electrocardiogram signals to estimate complex metrics such as age and gender. This study [5] employs deep learning for automatic food recognition systems. The results demonstrate that EfficientNet-V2 achieves performance close to the best-performing individual model on the MEFood dataset, while also having the lowest resource utilization and the shortest inference times. Clearly, computer-aided detection or diagnostic technologies [6, 7] provide significant momentum for the advancement of the biomedical field.

In the field of medical image segmentation, deep learning continues to demonstrate strong vitality [8, 9]. Convolutional Neural Networks (CNNs) [10] have successfully extracted image features, overcoming the limitations of traditional segmentation methods requiring manual feature extraction. As a classic architecture in deep learning, CNNs exhibit robustness to noise in medical images, enabling target recognition, feature extraction, and automatic segmentation. Long et al. [11] proposed the milestone Fully Convolutional Neural Network (FCN), replacing fully connected layers with convolutional layers and introducing transpose convolution, enabling the processing of images of any size and overcoming the limitation of CNNs requiring fixed input image sizes. However, FCN is insensitive to image details and does not consider relationships between pixels. The emergence of FCN has prompted extensive research into image segmentation algorithms, resulting in numerous segmentation models based on FCN improvements. Zhao et al. [12] introduced PSPNet, featuring a pyramid pooling module that aggregates context information from different regions to enhance the ability to obtain global information. Chen et al. [13] introduced DeepLabv3 + , which builds upon previous versions with multiple improvements. By incorporating key techniques such as dilated convolutions, multi-scale atrous spatial pooling, and a decoder, the model enhances the accurate segmentation performance for objects at various scales. Ronneberger et al. [14] introduced a symmetric U-shaped network, U-Net, connecting features of the same level between the encoder and decoder through skip connections to effectively fuse low-level and deep-level image features. Due to U-Net’s significant contribution to medical image segmentation, it quickly became a common benchmark, leading to the development of many improved U-Net-based segmentation models. Oktay et al. [15] applied attention mechanisms to the U-Net segmentation network, proposing Attention U-Net, which effectively focuses on salient regions and suppresses irrelevant background regions. Zhou et al. [16] introduced a nested U-Net architecture — UNet +  + , which improves image segmentation accuracy by adding connection modes and multi-scale feature fusion. Alom et al. [17] improved information propagation by introducing residual connections and a recurrent structure, proposing R2U-Net. Jafari et al. [18] propose DRU-Net, which integrates the strengths of ResNet and DenseNet, achieving higher segmentation accuracy. Lou et al. [19] proposed DC-UNet, which achieved significant performance improvements on challenging datasets by designing efficient CNN architectures to replace the encoder and decoder, and applying residual modules to replace skip connections. Some researchers have proposed lightweight medical image segmentation models [20–22], which demonstrate superior segmentation performance while reducing the number of parameters.

However, the aforementioned network models predominantly rely on CNNs and excel in capturing local features for long-term relationship modeling. Despite their proficiency in local feature extraction, CNN-based methods for segmentation tasks lack the ability to interpret long-term image correlations, thereby failing to extract global features. Inspired by the self-attention mechanism in Transformers [23] from the field of natural language processing, Dosovitskiy et al. [24] extended it to visual tasks, introducing the Vision Tansformer (ViT) that surpasses the limitations of CNNs. TransUnet, proposed by Chen et al. [25], was among the first studies to incorporate the Transformer structure into medical image segmentation. This model combines the strengths of CNNs in emphasizing local details and Transformers in globally modeling, providing a robust alternative for medical image segmentation. Cao et al. [26] introduced SwinUnet, a pure Transformer structure similar to U-Net for medical image segmentation. Marked image blocks are fed into a U-shaped Encoder-Decoder architecture based on Transformers through skip connections for local and global semantic feature learning.

Despite the widespread adoption of ViT in medical image segmentation to address the limitations of traditional CNNs in global context modeling, there are still significant constraints in terms of computational cost and learning fine-grained features. Xie et al. [27] proposed CoTr, which employs CNN for feature extraction and utilizes an efficient deformable Transformer to model long-range dependencies on the extracted feature maps. This approach significantly improves the accuracy and efficiency of 3D medical image segmentation while reducing computational and spatial complexities. Rehman et al. [28] introduced MaxViT-UNet, which effectively utilizes multi-axis self-attention mechanisms, allowing the model to focus on features at both local and global axes. This enhances discriminative ability between target and background regions, contributing to improved segmentation efficiency. Bian et al. [29] improved the self-attention mechanism in Transformers and introduced local multi-scale information to complement feature information. They proposed a Transformer-CNN Interactive (TCI) feature extraction module to build TCI-UNet, enabling the network to model global context information while paying attention to crucial local details. Wu et al. [30] introduced a Multiscale Efficient Transformer Attention (META) mechanism for rapid and high-precision polyp segmentation. In this approach, efficient transformer blocks are employed to generate multiscale element-wise attention. Chen et al. [31] proposed Patches Convolution Attention based Transformer UNet (PCAT-UNet), which is a U-shaped network based on a Transformer with a convolutional branch. It incorporates skip connections to fuse deep and shallow features from both sides. By leveraging the complementary advantages of both aspects, it can effectively capture global dependencies and details in the feature space of lower layers. Ibtehaz et al. [32] explored several advantages of Transformer-based U-Net models, particularly remote dependencies and cross-level skip connections. They attempted to simulate these aspects using convolutional operations and proposed ACC-UNet, a fully convolutional U-Net model that combines the inductive bias of CNNs and design decisions from Transformers. Its performance rivals that of Transformer-based models, such as SwinUnet or UCTransNet.

While the aforementioned segmentation methods can be applied to the segmentation of renal tumors, considering the inherent characteristics of renal tumors such as blurry boundaries, uncertainty in morphology, size, and location, some researchers have explored renal tumor segmentation methods based on deep features. Yu et al. [33] proposed Crossbar-Net, which captures both global and local appearance information of renal tumors from vertical and horizontal directions simultaneously. Through iterative training in a cascaded manner, two-directional sub-models are trained, complementing each other for self-improvement and ultimately achieving better segmentation performance. Sun et al. [34] introduced FR2PAttU-Net, incorporating R2Att networks and parallel convolutions to enhance the model’s width. Additionally, the model employs a fuzzy set enhancement algorithm to enhance adaptability to different image scale features, enabling high-precision tumor segmentation even in cases of unclear renal tumors. Hwang et al. [35] proposed RBCA-Net, which enhances segmentation performance through the use of a two-stage cascade approach. Hu et al. [36] presented TA-UNet3 + , replacing the encoder part of the neural network with a visual transformer and innovatively adding a new attention mechanism—Encoder-Decoder Transformer (EDformer)—to learn local features in skip connections.

However, existing models lack specificity in extracting features of renal tumors at different network hierarchical levels, leaving room for improvement in effectively and accurately segmenting renal tumors. To address this issue, we propose STC-UNet, a renal tumor segmentation method based on enhanced feature extraction. This method adapts to the unique features of renal tumors at different network levels, achieving highly accurate automatic segmentation of renal tumors in abdominal CT images. In this paper, we emphasize the following contributions:

- Unlike a simple approach of enhancing feature extraction, this paper focuses on the targeted enhancement of unique features at different network layers when extracting renal tumor features.
- This study represents a novel attempt to combine CNN and Transformer: integrating a non-patch implementation of ViT into a deep network with smaller feature maps and richer global features to enhance the extraction of semantic features in the deep network.
- Our model is validated on the KiTS19 dataset, showing improvements over the baseline model with increases of 1.60%, 2.02%, 2.27%, 1.18%, 1.52%, and 1.35% in IoU, Dice, Accuracy, Precision, Recall, and F1-score, respectively.
- Evaluation on CT images of actual renal tumor patients from the First Affiliated Hospital of Zhengzhou University demonstrates the superior segmentation performance and generalization ability of STC-UNet.

The remainder of this paper is organized as follows: Section II provides a detailed description of the network architecture proposed in this paper. Section III introduces the relevant settings of our experiments. Section IV conducts comprehensive experiments and visualization analysis. Section V discusses the effectiveness and impact of the proposed method. Finally, Section VI summarizes the entire work.

## Methodology

This paper proposes STC-UNet, a kidney tumor segmentation model that enhances feature extraction at different network levels. Based on the U-Net architecture, this model incorporates the SK module, the patch-free ViT module, and the coordinate attention mechanism to achieve precise segmentation of kidney tumors. The following sections will introduce the proposed model architecture and each of its modules in detail.

### STC-UNet

To achieve precise segmentation of renal tumors, this paper proposes an improved version of the U-Net model, named STC-UNet. In the U-Net network architecture, as the network layers deepen, the detailed information of the input image gradually diminishes, while the semantic information progressively increases. Therefore, our STC-UNet is based on enhanced feature extraction to accommodate the unique features of renal tumors at different network hierarchical levels.

**Fig. 1:**
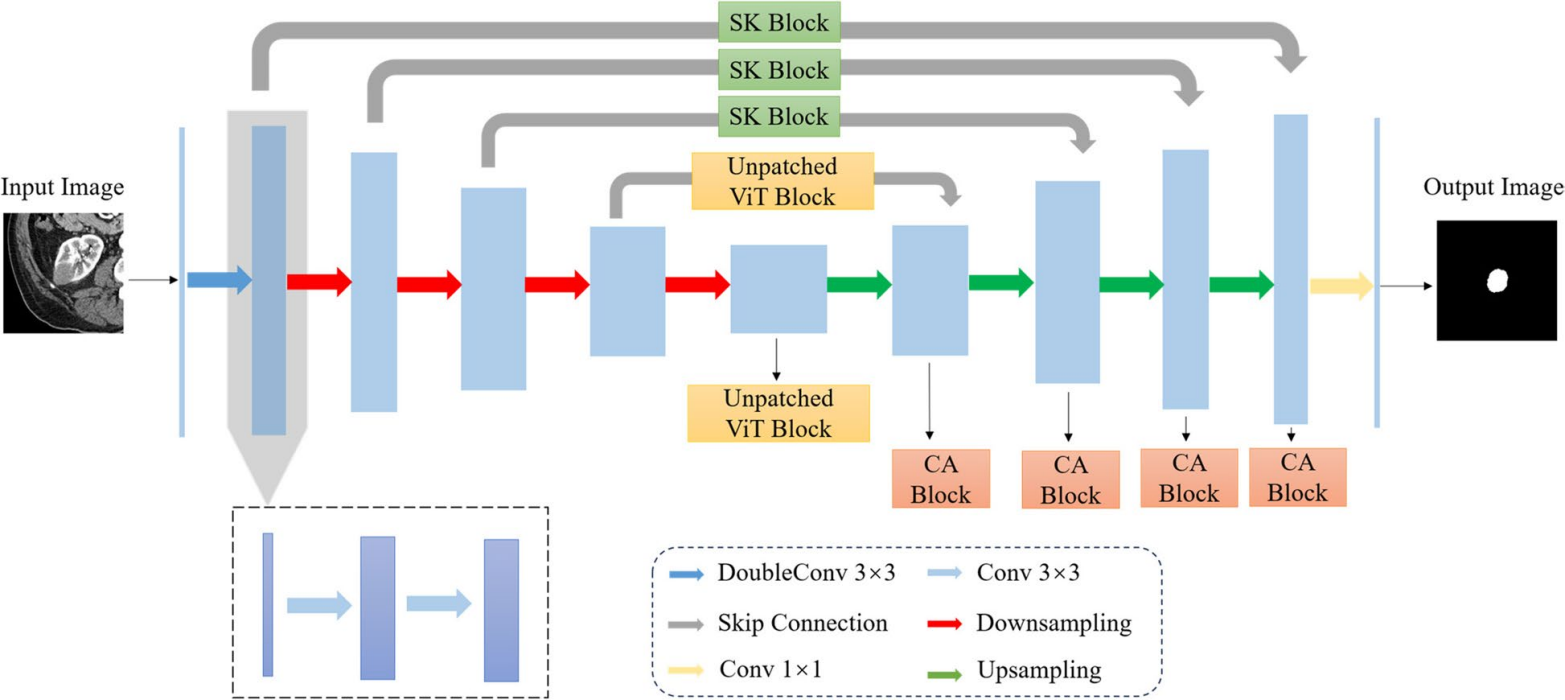
The network structure of STC-UNet. It is an improvement upon the U-Net model. In its shallow layers, specifically the skip connections in the first three stages, we incorporate the SK modules. In its deep layers, after the skip connections in the fourth stage and the double convolutions in the fifth stage, we introduce non-patch-based ViT modules. Additionally, in its decoder, we embed the CA modules

In this paper, the first, second, and third stages of the original U-Net model are defined as shallow layers, while the fourth and fifth stages are defined as deep layers. Capitalizing on the richness of image detail information in the shallow layers of the U-Net model, we introduce the Selective Kernel (SK) [37] module. By selectively utilizing convolutional kernels of different scales, the model can capture and retain these details at earlier layers, enhancing the extraction of multi-scale details of renal tumor features. Addressing the characteristics of the U-Net model, where the deep network exhibits rich semantic features and smaller-sized feature maps, we integrate a non-patch implementation of the ViT module into its deep network. It enables the model to capture long-range contextual information globally. To overcome the limitations of traditional ViT in local information modeling, its non-patch implementation facilitates pixel-level information interaction, aiding in capturing fine-grained local details. The non-patch implementation of the ViT module enhances global–local information synergy, thereby strengthening the model’s extraction of semantic features related to renal tumors. Finally, in the U-Net decoder section, the Coordinate Attention (CA) [38] mechanism is introduced. By embedding positional information into the channel attention mechanism, it enhances the model’s feature recovery and tumor region localization capabilities. The network structure of our proposed STC-UNet is illustrated in Fig. 1.

### Selective Kernel network

Renal tumors usually have richer detailed features, including gray scale distribution, homogeneity, margins, texture, density/intensity changes and other detailed information. By analyzing these detailed features, doctors and researchers can obtain more quantitative information about the tumor, such as the growth rate, malignancy degree, and prognosis of the tumor, which is important for tumor diagnosis and evaluation. During the encoding stage of U-Net, the downsampling process through pooling operation, the size of the feature map becomes smaller and lower resolution, which will lead to a part of the detail information is lost. During the decoding stage of U-Net, while the original image size can be recovered through the up-sampling operations, the lack of information from the encoding stage means that the simple jump connections employed during the up-sampling process do not fully leverage the tumor feature information in the feature map. This results in the recovered features lacking detailed information and edge sharpness.

**Fig. 2:**
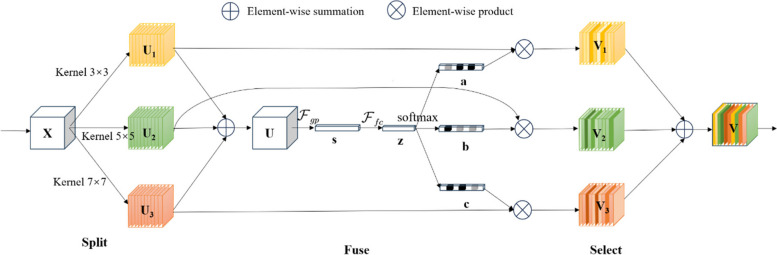
Selective Kernel Network. This structure consists of three branches, each equipped with convolutional kernels of sizes 3, 5, and 7, respectively

The U-Net shallow network produces high-resolution feature maps with rich detailed information. Therefore, this paper introduces the SK module into the U-Net’s shallow network, where detailed information is abundant. The SK module employs an innovative design by incorporating multiple-scale convolutional kernels and an attention mechanism to enhance the extraction of detailed features from renal tumors of various sizes and shapes. The network structure of the SK module is illustrated in Fig. 2, and it primarily consists of the following three steps:

- *Split*: The original feature map $X\in {\mathbb{R}}^{H\times W\times C}$ goes through three branches with convolutional kernel sizes of $3\times 3$, $5\times 5$, and $7\times 7$, respectively, to obtain new feature maps ${U}_{1}$*, *${U}_{2}$, and ${U}_{3}$.
- *Fuse*: Features from multiple branches are fused to obtain a feature map *U* with multiple sensory field information. feature map *U* is generated by global average pooling to embed global information $s\in {\mathbb{R}}^{C}$, and then *s* is passed through the fully-connected layer to obtain a compact feature $z\in {\mathbb{R}}^{d}$, which reduces the dimensionality to improve efficiency.
- *Select*: Multiple feature vectors *a*, *b*, and *c* processed by softmax are used to multiply channel-by-channel the feature maps ${U}_{1}$*, *${U}_{2}$, and ${U}_{3}$ extracted by multiple branches in the Split stage to get the feature maps ${V}_{1}$*,* ${V}_{2}$, and ${V}_{3}$ of the channel attention, respectively, and finally the feature maps ${V}_{1}$*, *${V}_{2}$, and ${V}_{3}$ of the channel attention are fused to get the final feature map *V* of the channel attention.

### Vision transformer

Renal tumors typically exhibit diverse semantic features, encompassing information such as tissue type, morphological structure, spatial distribution, and pathological regions. Accurately identifying and analyzing the semantic information of tumors can assist doctors in making more precise diagnoses. Although U-Net is capable of perceiving semantic features, its implementation still relies on convolution, leading to limited receptive fields. This limitation results in insufficient extraction of semantic features related to renal tumors.

**Fig. 3:**
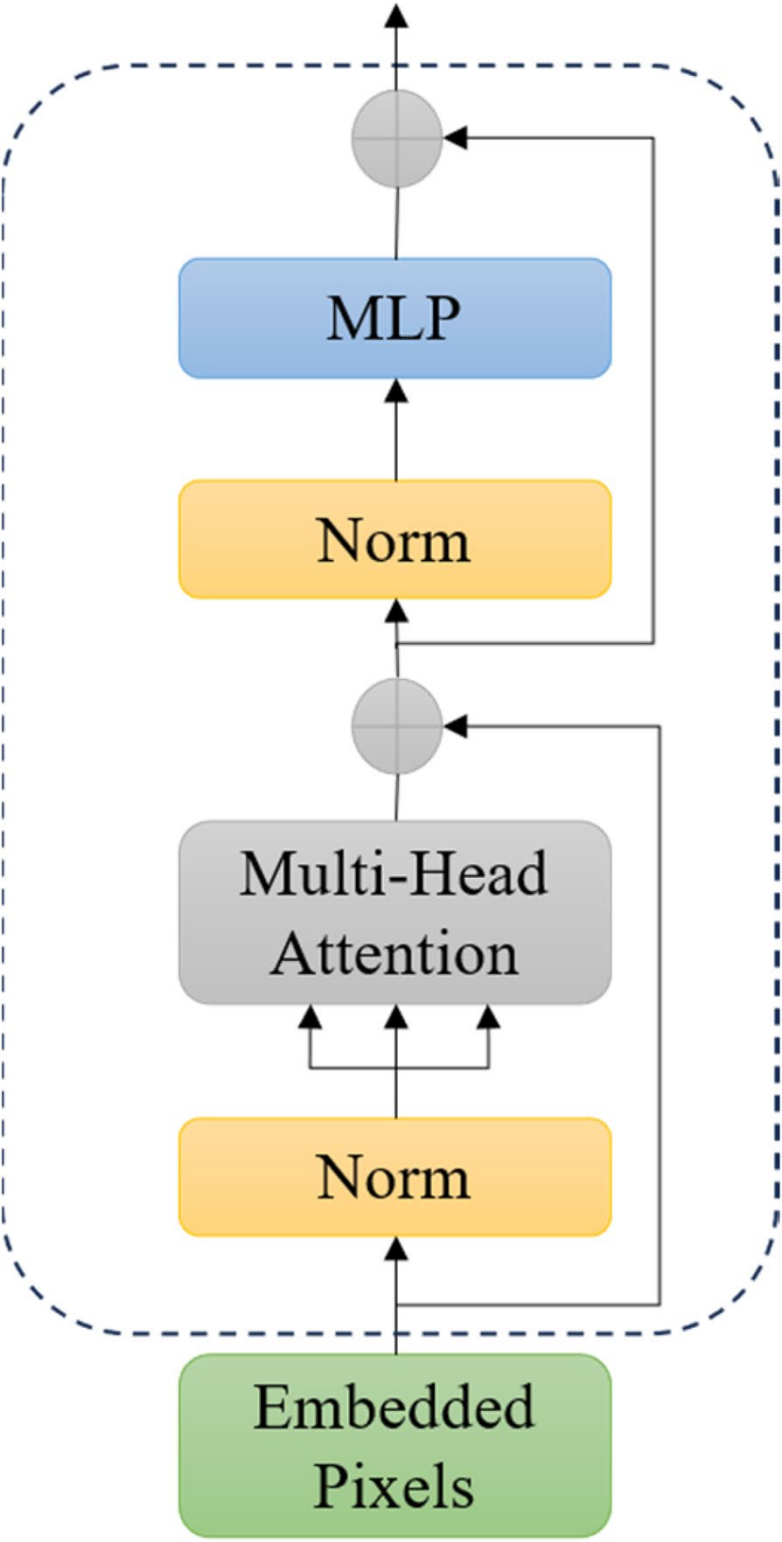
Network structure of vision transformer

To enhance the model’s long-range dependency modeling capability, a ViT module with global feature perception is introduced into the deep network of U-Net. In traditional ViT approaches, the original image is usually divided into fixed-sized blocks, which are then passed through the Transformer Encoder to extract features. However, this method may lose some fine-grained pixel-level information critical for tasks like renal tumor segmentation that require high precision. Using ViT in a non-patch manner involves directly inputting the entire image into ViT, making the input sequence length equal to the number of pixels in the input image. This allows self-attention interaction between pixels, addressing the limitation of traditional ViT in lacking local interaction information and preventing the loss of detailed features. While this approach introduces additional parameters and computational complexity, the reduced feature map size in the deep layers of U-Net significantly decreases the model’s computational demands and memory requirements compared to pixel-level processing of the original image. Therefore, this paper introduces the ViT module into the deep network of U-Net, enhancing the extraction of global features related to renal tumors. Additionally, the pixel-level information interaction facilitated by the non-patch implementation of ViT improves the extraction of local features. In summary, incorporating a non-patch ViT module into a deep network with rich semantic information contributes to the coordinated enhancement of global and local features, thereby strengthening the extraction of semantic features related to renal tumors. The network structure of the non-patch ViT module in this paper is illustrated in Fig. 3. The implementation principles and details can be divided into the following steps.

#### Pixel embedding

Since we use a non-patch implementation of the ViT module in this paper, the input sequence of the model will be a one-dimensional array composed of pixels from the image. As an example of embedding this module in the fourth layer of U-Net, the input image $X\in {\mathbb{R}}^{H\times W\times C}$ undergoes three downsamplings, resulting in a feature map with the shape ${X}_{4}\in {\mathbb{R}}^{\frac{H}{16}\times \frac{W}{16}\times 512C}$. Where $H\times W$ represents the resolution of the original image, and *C* represents the number of channels. Therefore, the effective sequence length input to the Transformer is $\frac{H}{16}\times \frac{W}{16}$. This sequence is then mapped to *D* dimensions using a trainable linear projection.

#### Position embedding

Since the transformer model does not have the ability to handle the positional information of the sequence, it is necessary to add positional encoding to each element of the sequence. The resulting sequence of embedding vectors serves as input to the encoder.

1 $${z}_{0}=\left[{x}_{class};{x}_{p}^{1}E;{x}_{p}^{2}E;\cdots ;{x}_{p}^{N}E\right]+{E}_{pos},E\in {\mathbb{R}}^{\left(1\cdot 1\cdot C\right)\times D},{E}_{pos}\in {\mathbb{R}}^{\left(N+1\right)\times D}$$

#### Transformer encoder

After feature embedding and positional embedding, the resulting feature sequence is fed as an input to the Transformer Encoder, which consists of multiple encoder layers, each containing a multi-head self-attention mechanism and a feed-forward neural network. Layer normalization (LN) is applied before every block, and residual connections are applied after every block. These layers are capable of global context modeling and feature representation learning of feature sequences.

2 $$z`_{\ell}=MSA(LN(z_{\ell-1}))+z_{\ell-1},\ell=1\,...\,L$$

3 $$z_{\ell}=MLP(LN(z`_{\ell}))+z`_{\ell},\ell=1\,...\,L$$

#### Multilayer Perceptron (MLP)

After a series of encoder layers, the feature representation of a Class Token is output, which is fed into the MLP module to output the final classification result.

4 $$y=LN({z}_{L}^{0})$$

### Coordinate attention

**Fig. 4:**
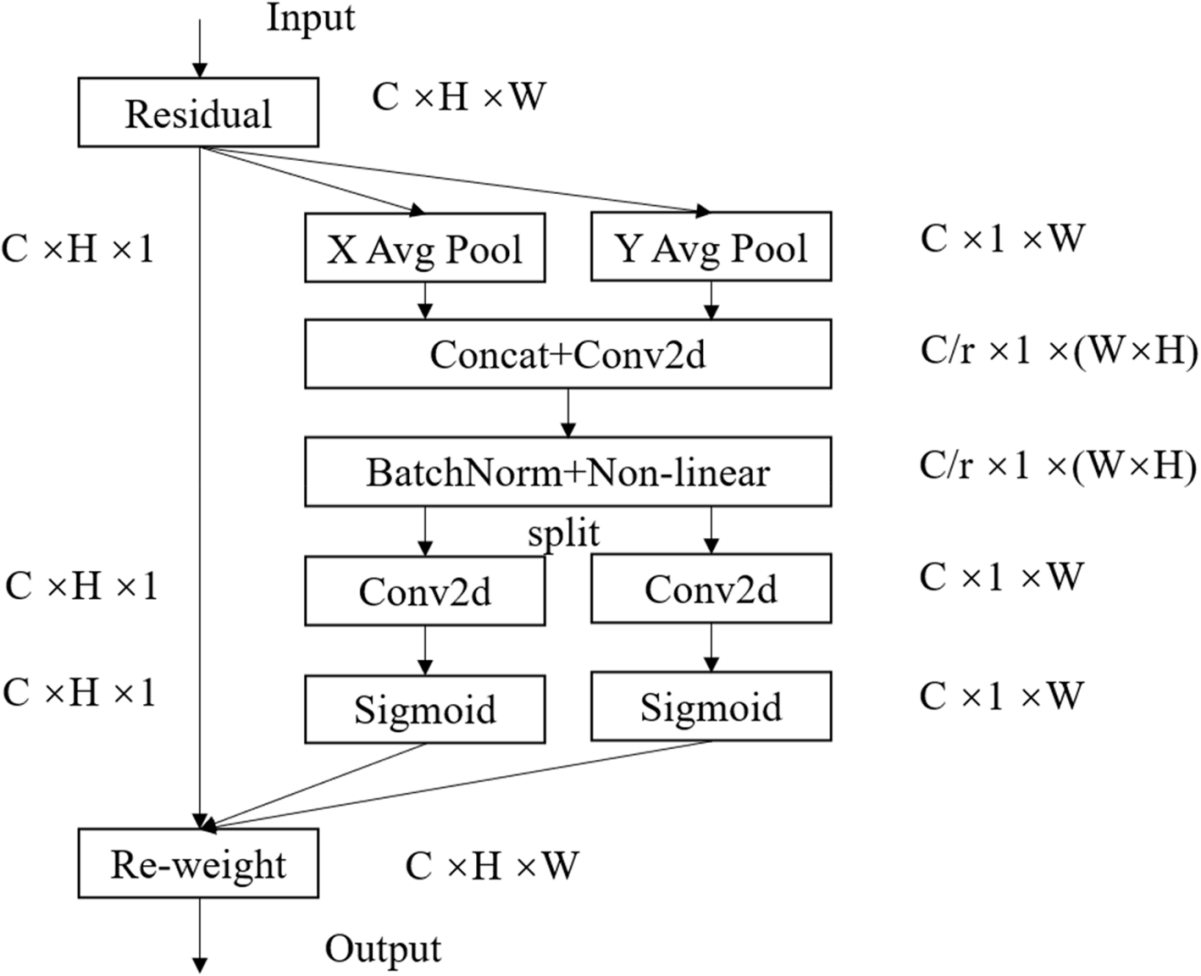
Network structure of coordinate attention

In the previous section, the U-Net model is improved by enhancing both the detailed features of renal tumors and semantic feature extraction, at which point the decoder of the model has adequately captured the feature information of renal tumors. However, in order to help the model locate the tumor region more accurately, the decoder needs to establish long-distance connections to better understand the correlation between channels and learn the spatial location information of different regions in the image. Therefore, in this paper, the CA module is added to the decoder part of the U-Net model to enhance feature recovery and tumor region localization capabilities, and its network structure is shown in Fig. 4.

The coordinate attention mechanism achieves precise encoding of positional information for channel relationships and long-range dependencies through two steps: embedding of coordinate information and generation of coordinate attention.

### Coordinate information embedding

Due to the difficulty in retaining positional information with global pooling in channel attention, the coordinate attention mechanism decomposes global pooling into horizontal and vertical directions. Specifically, given an input *X*, we encode each channel along the horizontal and vertical coordinates using pooling kernels with spatial extents of (*H*, 1) or (1, *W*) , respectively. Therefore, the output of the *c*-th channel at height *h* can be expressed as:

5 $$z_c^h\left(h\right)=\frac{1}{W}\sum\limits_{0 \leqslant i < W}x_c\left(h,i\right)$$

Similarly, the output of the *c*-th channel at width *w* can be expressed as:

6 $$z_c^w\left(w\right)=\frac{1}{H}\sum\limits_{0 \leqslant j < H}x_c\left(j,w\right)$$

The aforementioned transformations aggregate features along two spatial directions, generating a pair of direction-aware feature maps. This allows the attention block to capture long-range dependencies along one spatial direction while retaining precise positional information along the other, thereby aiding the network in more accurately localizing the objects of interest.

### Coordinate attention generation

In the coordinate attention generation phase, the global receptive field is utilized to encode precise positional information. Specifically, the aggregated feature maps generated by Eqs. (5) and (6) are concatenated and then passed through a shared $1\times 1$ convolutional transformation function *F*1, resulting in:

7 $$f=\delta \left(F1\left(\left[{z}^{h},{z}^{w}\right]\right)\right)$$

Here, $\left[\cdot ,\cdot \right]$ denotes the concatenation operation along the spatial dimension, and $\delta$ represents a nonlinear activation function. We then split along the spatial dimension into two separate vectors ${f}^{h}$ and ${f}^{w}$, and apply two $1\times 1$ convolutional transformations ${F}_{h}$ and ${F}_{w}$ to ${f}^{h}$ and ${f}^{w}$ respectively, yielding:

8 $${g}^{h}=\sigma \left({F}_{h}\left({f}^{h}\right)\right)$$

9 $${g}^{w}=\sigma \left({F}_{w}\left({f}^{w}\right)\right)$$

The outputs ${g}^{h}$ and ${g}^{w}$are then expanded and used as attention weights. Finally, the output of the coordinate attention $Y$ is given by:

10 $${y}_{c}\left(i,j\right)={x}_{c}\left(i,j\right)\times {g}_{c}^{h}\left(i\right)\times {g}_{c}^{h}\left(j\right)$$

## Experimental setup

### Dataset

This study evaluates the performance of a model using the KiTS19 dataset from the 2019 Kidney Tumor Segmentation Challenge [39]. The dataset includes abdominal CT scan images from 210 patients, with manual annotations by experts for the segmentation labels of the kidney and tumor regions, all in NIFTI format.

To reduce the complexity of renal tumor segmentation and improve the accuracy of tumor segmentation, preprocessing is performed on the original CT images. Slicing is applied to the 3D data of each patient along the transverse plane, resulting in a series of 512 × 512-sized images. Subsequently, window width and window level adjustments are made to enhance the contrast between renal tumors and other tissues. Negative samples are excluded by removing slices that did not contain renal tumors. Furthermore, a region of interest is selected by cropping areas containing renal tumors from the effective 2D slices. The cropped images are then resampled, resizing them to a uniform size of 512 × 512 pixels.

The processed dataset divides 5327 images randomly into training (4262 images) and testing (1065 images) sets at an 8:2 ratio. During subsequent training, online data augmentation techniques, including random cropping, flipping, and color distortion, are employed to dynamically augment the input data, generating diverse training samples. This effectively alleviates overfitting and enhances the model’s generalization capabilities.

### Evaluation indicators

To assess the effectiveness of our proposed method, we employ common objective evaluation metrics, including Intersection over Union (IoU), Dice, Accuracy, Precision, Recall, and F1-score, to evaluate the model’s segmentation performance on renal tumors. The values of these metrics range from 0 to 1, with larger values indicating better segmentation performance of the model.

The IoU represents the ratio of the intersection to the union between the predicted sample and the actual sample, as expressed in Eq. (11):

11 $$IoU=\frac{TP}{FN+FP+TP}$$

The Dice coefficient is a similarity measure for sets, commonly used to calculate the similarity between two samples, as defined in Eq. (12):

12 $$Dice=\frac{2\times TP}{2\times TP+FN+FP}$$

Accuracy represents the percentage of correctly predicted samples out of the total samples, as shown in Eq. (13):

13 $$Accuracy=\frac{TP+TN}{TP+FP+TN+FN}$$

Precision represents the proportion of true positive samples among all samples predicted as positive by the model, as shown in Eq. (14):

14 $$Precision=\frac{TP}{TP+FP}$$

Recall denotes the proportion of all samples with positive true labels that the model successfully predicts as positive, as in Eq. (15):

15 $$Recall=\frac{TP}{TP+FN}$$

The F1-score represents the harmonic mean of precision and recall, as shown in Eq. (16):

16 $$F1-score=2\times \frac{Precision\times \text{Re}call}{Precision+\text{Re}call}$$

where *TP*, *TN*, *FP*, *FN* represent the number of renal tumor pixels that are classified correctly, the number of background pixels that are classified correctly, the number of renal tumor pixels that are classified incorrectly, and the number of background pixels that are classified incorrectly in the CT images, respectively.

### Implementation details

In our experiments, our model is trained using the Adam optimizer with a learning rate set to 0.00001, a batch size of 4, and 50 epochs. We train all models on the NVIDIA GeForce RTX 4090 (24 GB) Graphics Processing Unit (GPU), and the same settings and training strategies are applied.

In CT slice images of renal tumors, as renal tumors usually occupy only a small portion of the image, the majority of pixels belong to non-tumor regions. The actual tumor region is relatively small, leading to a significant class imbalance issue. To address this problem, we employ a composite loss function composed of a dice loss and binary cross-entropy loss. The formula is as follows:

17 $$Total\ Loss=Dice\ Loss+BCE\ Loss$$

where Dice Loss and BCE Loss represent the dice loss and binary cross-entropy loss, respectively. Their formulas are as follows:

18 $$Dice\ Loss=1-\frac{2\sum\limits_{i=1}^{N}\left({y}_{i}{p}_{i}\right)+\in }{\sum\limits_{i=1}^{N}\left({y}_{i}+{p}_{i}\right)+\in }$$

19 $$BCE\ Loss=-\frac{1}{N}\sum\limits_{i=1}^{N}\left[{y}_{i}\cdot \text{log}\left({p}_{i}\right)+\left(1-{y}_{i}\right)\cdot \text{log}\left(1-{p}_{i}\right)\right]$$

where ${y}_{i}$ represents the true value of the *i*-th pixel, ${p}_{i}$ represents the predicted value of the *i*-th pixel. *N* is the total number of pixels, and $\varepsilon$ is a smoothing value to prevent division by zero issues.

## Experimental results

### Ablation studies

In this study, we extend the baseline U-Net model by incorporating three additional modules: SK, ViT, and CA. To investigate the impact of these modules on the segmentation performance of the proposed method, a series of ablation experiments are conducted in this section. The effects of each module on the model performance are assessed using evaluation metrics. Sequentially, the SK module, ViT module, and CA module are added to the baseline model, and the experimental results are presented in Table 1. The table indicates that with the addition of each module, the segmentation performance of the model improved to varying degrees. Moreover, when all modules are integrated, the model achieves optimal segmentation performance. This observation affirms the effectiveness of the three proposed modules in enhancing the segmentation capabilities of the model.

**Table 1:** Ablation experiments

U-Net	SK	ViT	CA	IoU(%)	Dice(%)	Accuracy(%)	Precision(%)	Recall(%)	F1-score(%)
√				95.80	94.92	94.27	97.05	96.18	96.61
√	√			96.34	95.62	95.49	97.16	96.99	97.08
√	√	√		97.13	96.60	96.33	97.92	97.56	97.74
√	√	√	√	**97.40**	**96.94**	**96.54**	**98.23**	**97.70**	**97.96**

### Comparison with state-of-the-arts

To validate the superiority of STC-UNet, a comparative experiment is conducted by inputting test images into pretrained U-Net [14], PSPNet [12], Deeplabv3 + [13], UNet +  + [16], DC-UNet [19], TransUnet [25], SwinUnet [26], MaxViT-UNet [28], and the proposed model. For an unbiased assessment of the impact of different methods on renal tumor segmentation, multiple evaluation metrics are employed, as shown in Table 2. The introduction of the SK module, with its multiscale convolutional operations, feature fusion, and selection mechanism, results in an increase in the parameter count. Additionally, the non-patch-based ViT module typically involves a higher number of parameters, resulting in an increased computational complexity and reduced processing speed. Thanks to the dual-channel CNN architecture, DC-UNet boasts a reduced parameter count of 10.81 million and achieves a high model FPS (frames per second) of 76.28. The multi-axis self-attention mechanism in MaxViT-UNet enables spatial interaction of local and global information, resulting in optimal Accuracy and Recall of 97.23% and 98.15%, respectively. It exhibits commendable segmentation performance, albeit with some limitations in precise boundary segmentation. In comparison to the baseline U-Net model, the proposed STC-UNet model demonstrates improvements in IoU, Dice, Accuracy, Precision, Recall, and F1-score by 1.60%, 2.02%, 2.27%, 1.18%, 1.52%, and 1.35%, respectively. Furthermore, when compared to several mainstream segmentation models, our proposed model achieves the optimal values for IoU, Dice, Precision, and F1-score. Therefore, the experimental results suggest that the proposed model possesses a certain degree of superiority.

**Table 2:** Segmentation results of different models on the KiTS19 dataset

Method	IoU(%)	Dice(%)	Accuracy(%)	Precision(%)	Recall(%)	F1-score(%)	Params(M)	FPS(img/s)
U-Net [14]	95.80	94.92	94.27	97.05	96.18	96.61	13.40	35.07
PSPNet [12]	95.33	94.30	95.06	95.71	96.71	96.20	46.58	31.89
Deeplabv3 + [13]	95.97	95.14	95.24	96.69	96.83	96.76	41.20	32.26
UNet + + [16]	96.40	95.69	94.85	97.69	96.57	97.12	39.96	33.36
DC-UNet [19]	94.47	93.14	95.34	94.06	96.89	95.43	**10.81**	**76.28**
TransUnet [25]	90.94	87.98	87.97	91.99	91.98	91.98	67.87	50.85
SwinUnet [26]	93.39	91.62	93.03	93.52	95.35	94.42	41.55	32.32
MaxViT-UNet [28]	97.13	96.61	**97.23**	97.33	**98.15**	97.74	27.26	18.72
**STC-UNet**	**97.40**	**96.94**	96.54	**98.23**	97.70	**97.96**	41.06	27.79

**Fig. 5:**
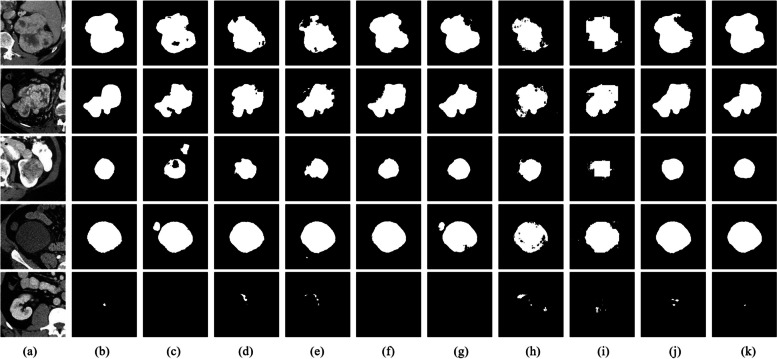
Visual Segmentation Results of Various Models on the KiTS19 Dataset. **a** is original image, **b** is the Ground truth, **c** is the output of U-Net, **d** is the output of PSPNet, **e** is the output of Deeplabv3 + , **f** is the output of UNet +  + , **g** is the output of DC-UNet, **h** is the output of TransUnet, **i** is the output of SwinUnet, **j** is the output of MaxViT-UNet, **k** is the output of Our STC-UNet

To further demonstrate the segmentation effectiveness of our proposed method on renal tumors, five images are selected randomly from the test set for visualization. As shown in Fig. 5, due to factors such as varying tumor sizes, diverse morphologies, indistinct boundaries, and interference from adjacent tissues, each model exhibits distinct segmentation results for renal tumors. For the first two images, most models demonstrate incomplete segmentation and rough contours in the tumor regions. In the third and fourth images, where tissues with colors and textures similar to tumors are present, U-Net struggles to differentiate effectively. In the fifth image, smaller tumors result in instances of under-segmentation by U-Net, UNet +  + , and DC-UNet, while PSPNet, DeepLabv3 + , TransUnet, SwinUnet, and MaxViT-UNet exhibit cases of over-segmentation and mis-segmentation. Meanwhile, the STC-UNet proposed in this paper exhibits a more comprehensive extraction of detailed and semantic features related to renal tumors. It places greater emphasis on both local and global information of the tumors, enabling a more precise segmentation of the renal tumor region. In summary, the segmentation performance of our model surpasses that of several other models, making it better suited for the task of renal tumor segmentation.

### Model generalization verification

**Fig. 6:**
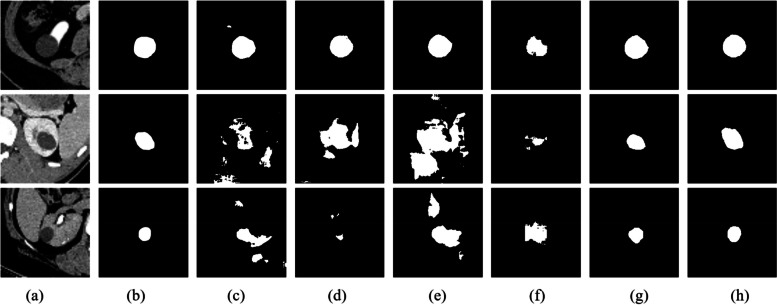
Visual Segmentation Results of Various Models on the Renal Tumor Dataset from the First Affiliated Hospital of Zhengzhou University. **a** is original image, **b** is the Ground truth, **c** is the output of U-Net, **d** is the output of UNet +  + , **e** is the output of DC-UNet, **f** is the output of SwinUnet, **g** is the output of MaxViT-UNet, **h** is the output of Our STC-UNet

In order to validate the effectiveness of STC-UNet in segmenting renal tumors and assess its generalization capability, we collaborate with the First Affiliated Hospital of Zhengzhou University to acquire abdominal CT images from 10 patients with renal tumors. All data are annotated for renal tumors in CT images under the guidance of professional radiologists specialized in medical imaging, and the annotations are ultimately verified through their examination. Our STC-UNet is then tested on this dataset, and the results are compared with U-Net [14], UNet +  + [16], DC-UNet [19], SwinUnet [26], and MaxViT-UNet [28]. The evaluation metrics are presented in Table 3. It can be observed that compared to other models, DC-UNet achieves the highest Accuracy and Recall, while our proposed STC-UNet model achieves optimal values for IoU, Dice, Precision, and F1-score. Subsequently, we select three CT image cases randomly for visualization, as illustrated in Fig. 6, to showcase the segmentation performance of various models under different conditions. It is evident that U-Net, UNet +  + , DC-UNet, and SwinUnet exhibit subpar segmentation results, whereas MaxViT-UNet and STC-UNet demonstrate superior segmentation performance. While DC-UNet provides comprehensive coverage of renal tumors, it also exhibits instances of erroneously segmenting some background regions as tumors, which explains its high Accuracy and Recall. MaxViT-UNet achieves good segmentation results, but its ability to delineate boundaries is comparatively weaker. In summary, our STC-UNet model outperforms other models in terms of segmentation effectiveness and showcases superior generalization capabilities.

**Table 3:** Segmentation results of various models on the renal tumor dataset from the first affiliated hospital of Zhengzhou University

Method	IoU(%)	Dice(%)	Accuracy(%)	Precision(%)	Recall(%)	F1-score(%)
U-Net [14]	85.90	79.18	81.89	84.50	87.93	86.12
UNet + + [16]	86.72	80.74	79.44	88.07	86.29	87.16
DC-UNet [19]	85.74	78.87	**92.06**	80.34	**94.71**	85.91
SwinUnet [26]	84.10	75.55	76.49	83.09	84.33	83.70
MaxViT-UNet [28]	89.49	85.62	89.85	87.99	93.23	90.41
**STC-UNet**	**89.85**	**86.23**	84.34	**92.17**	89.56	**90.82**

## Discussion

To address the segmentation challenges posed by the unclear boundaries, variable morphology, size, and position of kidney tumors, this paper proposes an improved version of U-Net, named STC-UNet, designed for precise kidney tumor segmentation by enhancing feature extraction at different network levels. It is well-known that in the U-Net architecture, as the network depth increases, the detailed information of the image gradually decreases while the semantic information increases. STC-UNet aims to enhance feature extraction to capture unique features at different network levels. First, we introduce the SK module into the shallow network of U-Net, where detailed features are abundant. The selection module within the SK module adaptively decides which scales of convolutional kernels to use on each channel. By selectively applying different scales of convolutional kernels, the SK module enhances the representation capabilities of features, thereby capturing multi-scale features at various levels. The SK module in the shallow network captures rich detailed and multi-scale features, addressing the challenges of unclear boundaries and uncertain sizes of kidney tumors.Second, we integrate the ViT module without patch splitting into the deep network of U-Net, where semantic features are abundant. The ViT module, with its inherent properties, effectively captures the global context information in the image. By not splitting patches in the deep network where the feature map is smaller, it reduces the loss of detailed information, compensating for the ViT module’s limitation in handling local information. The ViT module in the deep network captures rich semantic features while reducing the loss of detailed information, further enhancing the model’s ability to extract complex features, thereby better addressing the challenges posed by the unclear boundaries, morphology, size, and position of kidney tumors. Finally, combining the strong context information capturing capabilities brought by the SK and ViT modules, we introduce the coordinate attention mechanism in the decoder part, enabling more accurate feature capture of the tumor region, thereby further improving the precision and accuracy of segmentation.

Our STC-UNet’s computational efficiency is worth discussing. The introduction of Transformer modules does increase the computational complexity of our model. However, we only introduce Transformers in the deep layers with small feature maps, minimizing the impact on overall computational load. This design of the ViT module without patch splitting leverages the advantages of Transformers in extracting high-level features while reducing the loss of local details and, to some extent, decreasing computational complexity. Additionally, according to our experimental results, STC-UNet, as the optimal segmentation model, achieved an inference speed of 27.79 FPS. Although this is slightly below the widely accepted real-time segmentation standard of 30 FPS, considering that kidney tumor segmentation requires high precision rather than strict real-time performance, we believe this is acceptable. Despite performing efficiently on high-end GPUs, STC-UNet’s performance on CPUs is significantly slower, making it less suitable for real-time applications in low-resource settings. Future work could focus on model optimization techniques, such as model pruning, quantization, and the use of more efficient Transformer variants, to enhance the computational efficiency of STC-UNet.

## Conclusion

In this paper, we propose an improved version of U-Net, named STC-UNet, for kidney tumor segmentation. This is a segmentation network based on enhanced feature extraction for different network levels. Compared to other advanced 2D medical image segmentation models, our STC-UNet achieves higher accuracy and superior segmentation performance. On the KITS19 dataset, the Dice coefficient for kidney tumors reaches 96.94%, IoU reaches 97.40%, and Precision reaches 98.23%. Next, we apply STC-UNet to CT images of real patients with kidney tumors at the First Affiliated Hospital of Zhengzhou University. The experimental results show that STC-UNet exhibits a certain level of robustness and generalization capability, demonstrating favorable segmentation outcomes.

However, this study has several limitations. Firstly, the computational efficiency of STC-UNet is compromised due to the integration of complex modules, which may limit its applicability in real-time scenarios. Secondly, the generalization capability of STC-UNet needs further validation on larger and more diverse datasets to ensure its robustness across different clinical settings. Future work could focus on optimizing the computational efficiency of STC-UNet and exploring its performance on a wider range of datasets.

## Data Availability

Our code is available at: https://github.com/ahuweia/STC-UNet.git.
